# Study of medicinal plants used in ethnoveterinary medical system in riverine areas of Punjab, Pakistan

**DOI:** 10.1186/s13002-024-00686-9

**Published:** 2024-05-07

**Authors:** Muhammad Umair, Muhammad Altaf, Taswar Ahsan, Rainer W. Bussmann, Arshad Mehmood Abbasi, Mansour K. Gatasheh, Mohamed Elrobh

**Affiliations:** 1 College of Life Sciences, Zhejiang Normal University, Jinhua, 321004 China; 2 College of Life Sciences, Zhejiang University, Hangzhou, 310058 China; 3 Department of Forestry, Range and Wildlife Management, The Islamia University of Bahawalpur, Bahawalpur, 63100 Pakistan; 4 Institute of Plant Protection, Liaoning Academy of Agricultural Sciences, Shenyang, 110161 P.R. China; 5 Department of Ethnobotany, Institute of Botany and Bakuriani Alpine Botanical Garden, Ilia State University, 0105 Tbilisi, Georgia; 6 Staatliches Museum Für Naturkunde, Erbprinzenstrasse 14, 76133 Karlsruhe, Germany; 7 Department of Environment Sciences, COMSATS University Islamabad, Abbottabad Campus, Abbottabad, 22044 Pakistan; 8 Department of Biochemistry, College of Science, King Saud University, P.O. Box 2455, 11451 Riyadh, Saudi Arabia

**Keywords:** Ethnoveterinary remedies (EVR), Endo- and ecto-parasitic ailments, Disease cured level, Medicinal plants, Fidelity level, Principal component analysis

## Abstract

**Background:**

The use of medicinal plants to treat various veterinary illnesses has been practiced for millennia in many civilizations. Punjab is home to a diverse ethnic community, the majority of whom work in dairy farming, agriculture, and allied professions and have indigenous practices of treating animal illnesses using native flora. This study was designed to (1) document and preserve information about the applications of medicinal plant species in ethnoveterinary remedies among inhabitants of Punjab, Pakistan, and (2) identify popular plants for disease treatment by quantitative analysis of the obtained data and to assess the pharmacological relevance of these species.

**Methods:**

To collect data from informants (*N* = 279), questionnaires and semi-structured interviews were used. The ethnoveterinary data were analyzed using principal component analysis, relative frequency citation, fidelity level, relative popularity level, and rank order priority.

**Results:**

A total of 114 plant species utilized in the ethnoveterinary medicinal system were found, which were divided into 56 families and used to treat 16 different illnesses. The Poaceae family, with 16 species, was the most common in the region. The most commonly employed growth form in herbal preparation was herb (49%). The most used part in ethnoveterinary remedies was leaves (35%), while powder was the most commonly used way for preparing ethnoveterinary remedies (51 applications). According to principal component analysis, the most typically used species in the research region were grasses. Five grasses (*Arundo donax, Desmostachya bipinnata, Eleusine indica, Hordeum vulgare,* and *Pennisetum glaucum*) showed a 100% FL value when used to treat diuretics, helminthiasis, digestive problems, fever, cough, worm infestation, indigestion, galactagogue, oral infections, and genital prolapse. The maximum value of disease cured level (DCL%) was recorded at 87.6% for endo- and ecto-parasitic ailments in the study area.

**Conclusion:**

This study demonstrates that medicinal plants play an important part in satisfying farmers’ animal healthcare demands, making it a feasible practice. The study also provides a wealth of knowledge regarding ethnoveterinary methods for further planning and application, providing an option for farmers who cannot afford allopathic therapy.

## Introduction

Traditional knowledge developed over time is influenced by elements of ancestral inheritance, intercultural connection, and interaction with the natural environment [1]. These facts explain the reasons for the variations of traditional knowledge among cultures, locations and ethnic groups [2, 3]. Studies conducted in various regions of the world described the existence of cultural variety in plant use knowledge and treatment systems [3]. Many societies have used medicinal herbs for treating various veterinary illnesses for centuries [4]. Traditional healers mostly rely on medicinal plants, which have long been known to be a rich source of phytonutrients in animals [5]. Traditional knowledge is transmitted orally from generation to generation, like all other traditional knowledge systems [6], and may disappear altogether [7] as a result of rapid social, environmental, and technological change [8], as well as the disappearance of cultural legacies masquerading as civilization [9]. In order to conserve the traditional knowledge before it is permanently lost, methodical research must be used to record it via systematic investigations [10, 11]. In addition, more surveys are needed in various sections of the nation, encompassing a broader range of sociocultural groups, with the goal of obtaining unique knowledge and cultural variations [12].

In Pakistan, the livestock sector contributes almost 56% of the value of agriculture and almost 11% of agricultural GDP [13]. This vital sector not only provides animal feed to the rural population, but it also contributes to poverty alleviation by providing draught power and employment to the people of this country. Pakistan is the 3rd largest milk-producer in the world, and its output is rising overtime, highlighting the importance of the buffalo [14]. Many Pakistani livestock producers are disadvantaged, and the majority of these farmers are unable to purchase modern allopathic drugs due to financial constraints [15], resulting in low animal production and health. In such cases, ethnoveterinary treatment may be supported as an alternative to modern medications [16], and it can help reduce poverty by allowing people to cure their animals using their own resources. Despite advancements in the pharmaceutical industry and the development of therapeutic agents, traditional indigenous medicine is still utilized in rural areas for the treatment of human [13] and cattle [17] ailments as well as the preservation of outstanding animal health in developing countries [9, 18]. The development of the livestock industry in Pakistan is hindered by a variety of challenges, including policy concerns, the increasing degradation of rangelands, unsanitary eating habits, subpar marketing schemes, inadequate extension services, and a lack of resources [19]. In Pakistan, various animal illnesses can be deadly [20], include black quarter, bovine viral diarrhea, hemorrhagic septicemia, and foot and mouth diseases. Dairy productivity is reduced because farmers do not routinely vaccinate their animals against these devastating illnesses [21]. Some animals, for instance, appear to have mastitis, which significantly contributes to the decrease in milk output. While they can be fairly complicated, the effects of animal illnesses are typically only thought of as having direct effects. Diseases reduce animal output and take away potential daily income from producers. These debilitating illnesses cause morbidity, which causes temporary or permanent product loss [22]. Parasitism in the Modern World Camelids, which comprise both ecto- and endoparasites, is a major public health concern across the world [23], notably in developing countries such as Pakistan [24–28]. The hot humid climate in the riverine areas of Punjab province substantially encourages the growth and development of endo- and ectoparasites, which causes parasitism to be violent. Ecto- and endoparasite illnesses reduce feed intake and utilization efficiency as a result of preclinical or severe infections [29–31]. Ticks, lice, flies, mange, and mites are examples of ectoparasites that cause illness in animals, such as acute inflammation, irritation, hair loss, weight loss, dull body, anemia, skin damage, serum exudation, and crust development owing to serum exudate buildup. Death occurs in animals that are neglected and mistreated animals [32].

The documentation of EVR knowledge about the usage of medicinal plants by indigenous peoples is beneficial not only for the preservation of traditions, cultures, diversity of plant species but also for the development of drug and the protection of community healthcare in future [9]. Documenting EVR knowledge and evaluating plant uses for a range of purposes are important not only to preserve it, but also to make it available for future use in the face of fast cultural and socioeconomic and changes [33]. The main purpose of this study was to describe the medicinal applications of plant species used by the indigenous people of Punjab province for the treatment of veterinary health disorders. The main objectives were to (i) document the traditional EVR knowledge about the pharmacological uses of plant species and (ii) gather information on traditional treatments for a range of diseases, including the plant parts used, application, and preparation techniques.

## Materials and methods

### Study area

**Fig. 1:**
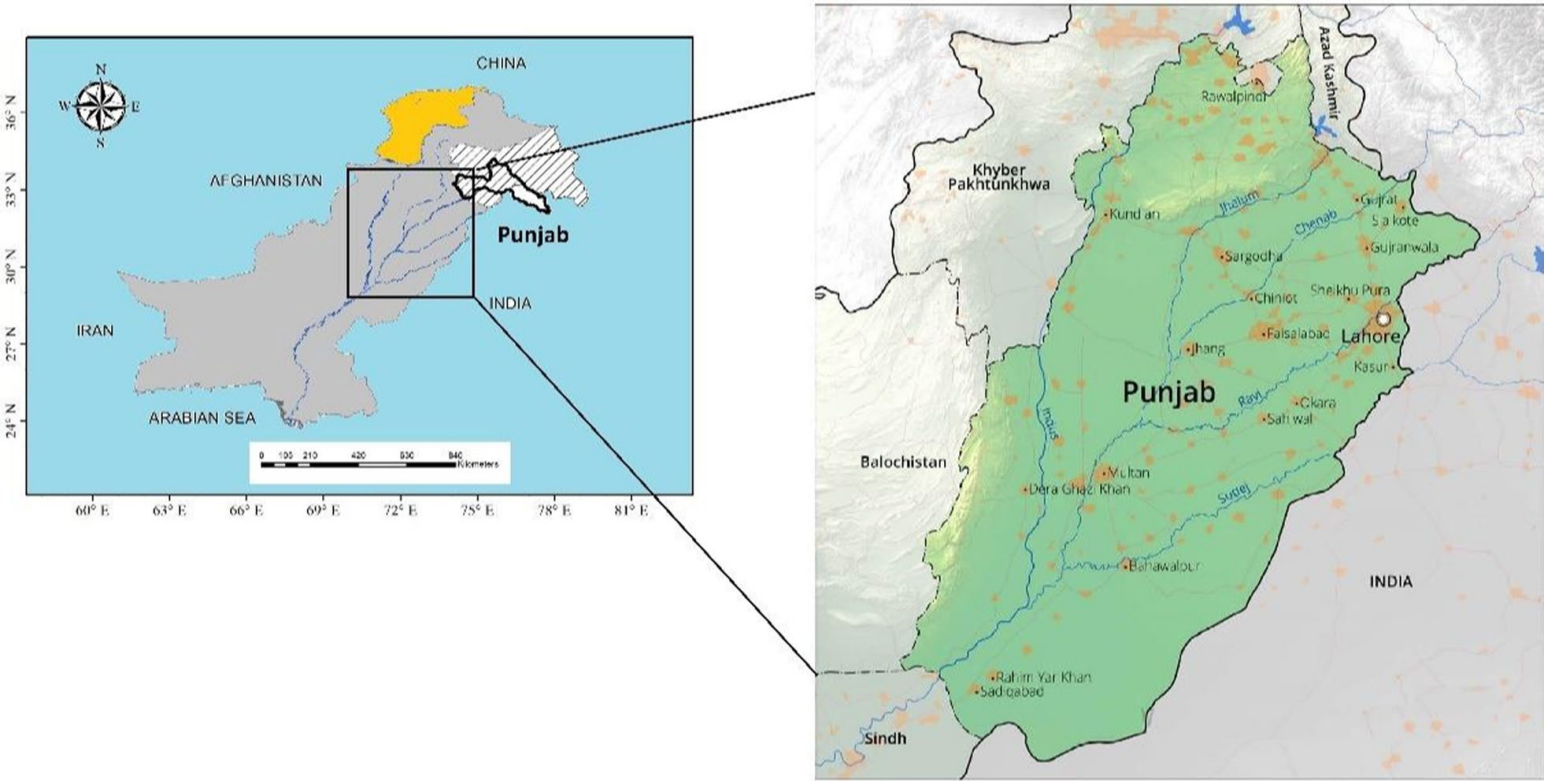
Map of Punjab showing study areas, i.e., river Chenab, river Sutlej, river Ravi, river Jhelum, and river Indus

This research was carried out in reverie areas of Punjab, Pakistan. It is located at latitudes ranging from 27° 42′ N to 34° 02′ N and longitudes ranging from 69° 81′ E to 75° 23′ E. The majority of Punjab is made up of lush alluvial plains that are intensively watered by five rivers: the Indus, Ravi, Jhelum, Chenab, and Sutlej (Fig. 1). Small desert regions may be found in the Suleiman Range and southern Punjab. Punjab province is the 2nd largest province in Pakistan in terms of terrain area, and the first in terms of population. Punjab produces a variety of seasonal crops, wheat, and rice as well. Certain cities in Punjab are highly inhabited. Punjab has an area of approximately 205,000 km^2^ and has elevations ranging from 200 to 2000 m. Both precipitation and temperature fluctuate throughout the year. The soil in the research region is mostly clay, loamy, and sandy [34]. Most of the places have foggy weather in the winter and scorching weather in the summer, with an average yearly temperature ranging from − 2 to 48 °C. The warmest month is June, while the coldest month is January. The annual mean rainfall over the previous 5 years has been around 479.8 mm. In comparison with the drier sections of the province, the northern regions of the province receive a good quantity of rainfall throughout the year [35].

The ethnic composition of the region is highly diversified, with several tribes and clans. The largest ethnic groups are Arain, Butt, Gujjar, Mughal, and Rana. The majority of population speaks Urdu and Punjabi, with Saraiki coming in second. At government offices, English is employed. Compared to the other provinces, Punjab has the greatest literacy rate. Also, Punjab province provides a significant portion of Pakistan’s GDP. The economy of this province is focused on farming, and wheat is the most extensively farmed crop, with substantial output of cotton, rice, sugarcane, maize, and grains. Farming is the primary employment of rural populations, and they rely on farming methods and farm animal’s management for a living. Because of linguistic and cultural differences, the people of Punjab province have a wide range of traditional knowledge and rituals.

### Data collection and identification of plant and animal species

**Fig. 2:**
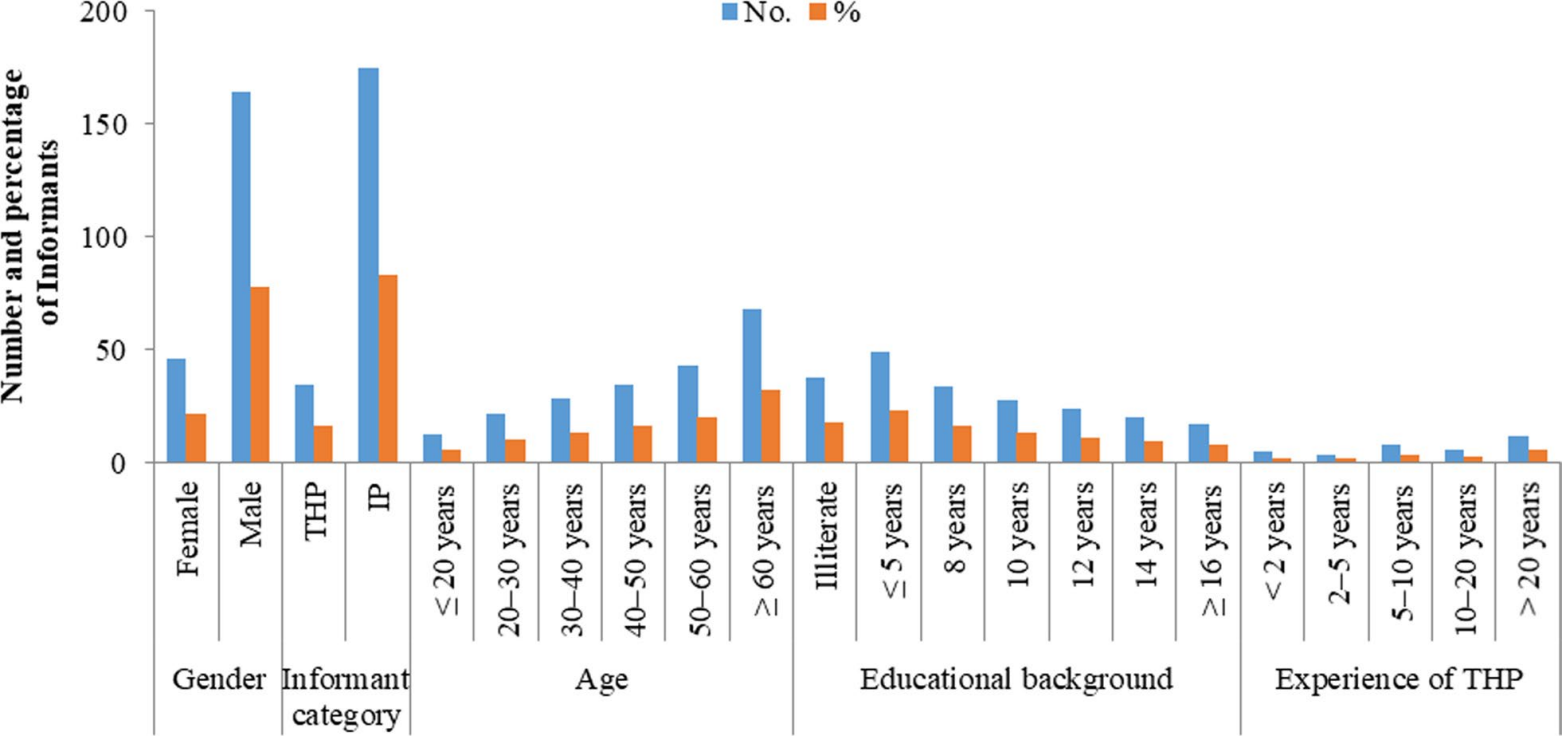
Demographic features of the study participants. Informant catagory is categorized as traditional health practitioners (THP) and indigenous peoples (IP)

This study was conducted between 2021 and 2022 with the objective of documenting medicinal plants used for treating livestock ailments. After obtaining oral prior-informed agreement, group discussions and interviews (semi-structured) were undertaken with 279 respondents to gather knowledge on the ethnoveterinary applications of plant species (Fig. 2). The “code of ethics” of the “International Society of Ethnobiology” was followed properly (http://www.ethnobiology.net/). Responses were recruited at random or, in certain cases, using the snowball approach [36, 37]. The surveys were initially developed in English before being translated into Urdu, Punjabi, and Saraiki. Demographic information on respondents included their gender, age, degree of education, and linguistic background was gathered.

The principal author of the study area is a resident who travelled with a photographer to both high and low elevated areas. Plants with therapeutic properties were gathered, and voucher specimens were submitted in the Department of Forestry Range and Wildlife Management, The Islamia University of Bahawalpur, Pakistan. During sampling, plant species were initially recognized, and the identifications were validated. The species entries were complemented along with data on taxonomic position (family), vernacular name, common name, growth form, and medicinal uses. The growth form was categorized into herbs, shrubs, grasses, climbers and trees according to the system proposed by [38, 39]. Collected plant species were identified by Prof. Dr. Tanveer Hussain (The Islamia University of Bahawalpur, Pakistan and by using The “Flora of Punjab” [40] and “Flora of West Pakistan” [41]. The Royal Botanical Gardens Kew Science (http://www.plantsoftheworldonline.org) and World Flora Online (http://www.worldfloraonline.org) websites were utilized to confirm the medicinal plant species taxonomy.

### Quantitative ethnoveterinary data analysis

The ethnoveterinary data were analyzed using various indices, including “Informant consensus factor” (ICF), disease cured level (DCL%), “fidelity level” (FL%), “relative popularity level” (RPL), and “rank order priority” (ROP). The index values are reported in proportions and percentages.

#### Disease cured level and informant consensus factor

Disease cured level (DCL%) describes the informants’ used percentage of medicinal plant species to cure a specific disease category. The maximum DCL% value indicates that there is homogeneity in the use of medicinal plants between the informants, whereas informant consensus factor (ICF) describes informants’ consensus on the medicinal plant consumption species and evaluates variability in mode of utilization against reported diseases. The maximum ICF value, i.e., close to 1, indicates that well-known species are used by a large proportion of local communities due to their authenticity regarding diseases. However, low ICF index close to 0 specifies that the informants use this species randomly to treat reported diseases [35].

Before calculating ICF and DCL value, ailments are broadly categorized into different categories, i.e., dermatological, endo- and ecto-parasitic, fever, gastrointestinal, reproductive, respiratory disorders, tonic, urinary disorders, and other health problem (hormonal disorders). The ICF and DCL% value was calculated using the following formula.

1 $${\text{ICF}}=\frac{{N}_{{\text{ur}}}-{N}_{{\text{t}}}}{{N}_{{\text{ur}}}-1}$$

2 $${\text{DCL}}\%=\frac{{N}_{{\text{ur}}}-{N}_{{\text{t}}}}{{N}_{{\text{total}}}}$$

where “*N*_total_” is the total number of citations for each disease category, “*N*_ur_” is the total number of use reports for each disease category, and “*N*_t_” indicates the number of taxa used in said category.

#### Relative popularity level (RPL)

“RPL” is the ratio of a plant medicinal use to the total number of informants for any sickness. Consequently, the healing abilities of plant species with similar FL values but different informant numbers may vary. As a result, a corrective scale was developed, and all of the observed species were categorized in accordance with their level of popularity. RPL index is a scale from 0 to 1 where “0” indicates that no ailments are treated by plant species and “1” indicates that plant species are completely popular for treating significant conditions. A popularity index would be at 1.0 when all species had an equal prevalence of main illnesses, and it would decrease to zero as the species’ relative popularity went away from the popular side [42]. Popular species’ RPL values were wisely set to be equal to 1. For plant species in an unpopular group, the RPL value is below one. Depending on their relative popularity level, the selected plant species for EVM are categorized as either unpopular or popular [43].

#### Fidelity level (FL%)

The “FL” is the percentage of participants in the study area who claim to have used a specific type of species [44]. The “FL” index was noted applying the formula by Friedman et al. [45],

3 $$\mathrm{FL\%}=\frac{N}{{N}_{p}}\times 100$$

where “*N*_*p*_” is the frequency with which interviewers indicated species for certain categories of medicinal use, and “*N*” denotes the total number of informants who mentioned the species for any reason. The high “FL” score reflected the relevance and regularity with which the research area’s respondents used the plant species for EVM.

#### Rank order priority (ROP)

With different FL and RPL values used as adjustment factors, species are suitably ordered using the “ROP” index [46–49]. The formula for “ROP” is to multiply RPL index by FL% [45]. The ROP value was obtained using the algorithm.

4 $${\text{ROP}}=\mathrm{FL }\times \mathrm{ RPL}$$

#### Statistical analysis

Using the ethnoveterinary data, multivariate ordination principal component analysis (PCA) was used to identify patterns of different growth forms of plants. The contribution of plant part usage in the preparation of ethnoveterinary remedies was displayed in chord diagrams using circlize package in R software (4.2.1). All ethnoveterinary data were analyzed using Microsoft Excel 2010 (Microsoft, Redmond, WA, USA), R software (4.2.1) and PAST 4.12b [50].

## Results

### Demographics of respondents

A total of 279 local informants were interviewed, comprising 231 men and 48 women (Fig. 2). These informants were divided into distinct classes based on demographic data as shown in Fig. 2. Local indigenous people (IP) accounted for 91 percent of the 279 respondents, compared to 9% of traditional medical practitioners (TMPs). Farmers, traditional healers or hakims, teachers, shopkeepers, and housewives were among the participants. The ages of the informants ranged from 19 to 70 years. Most interviewees (28%) were between the ages of 60 and 70 and had extensive traditional knowledge, whereas young informants provided minimal information. A total of 109 informants (39.1%) were illiterate, while the other informants had varying levels of education, including: less than “5 years of education” (31.9%), “8 years of education” (12.2%), “10 years of education” (9.68%), “12 years of education” (2.51%), “14 years of education” (2.87%), and more than “16 years of education” (1.79%).

### Useful plants

The usage of medicinal plants was frequently found to be a daily ritual in many homes, where such knowledge was passed down to the younger generation simply by observing the elders practice. There have been reports of 114 medicinal plant species from 49 families (Tables 1 and 2). Poaceae was the most dominant family of plants, with 12 species, followed by Solanaceae, Fabaceae, Euphorbiaceae and Asteraceae (6 species each), Apiaceae and Myrtaceae (5 species each), Malvaceae, Mimosaceae, and Moraceae (4 species each), Cucurbitaceae, Brassicaceae and Apocynaceae (3 species each), whereas other families connected with mainly two or fewer species (Table 2).

**Table 1:** Catalogue of medicinal plants used by the indigenous people of Punjab for ethnoveterinary practices

Sr. Nos	Species name and voucher no	Family	Habit	Common name	Local name	Diseases cured	Plant parts	Administration method	Used	N	Np	FL	RPL	ROP
1	*Acacia modesta* Wall* (EV-60)*	Mimosaceae	T	Amritsar gum	Phulai	Expulsion of placenta	Bark and Stem	Decoction	Oral	33	8	25	0.6	15
						Easy delivery								
2	*Acacia nilotica* (L.) Delile. *(EV-61)*	Mimosaceae	T	Arabic gum	Kekar	Genitals prolapse	Barks	Powder, Decoction	Oral	25	6	25.64	0.61	16
						Worm infestation								
3	*Achyranthes aspera* L.* (EV-2)*	Amaranthaceae	S	Prickly-chaff flower	Puth Kanda	Ectoparasites	Leaves	Powder	Topical	39	13	33.33	0.79	26
						Nasal myiasis								
4	*Allium cepa* L.* (EV-50)*	Liliaceae	H	Onion	Piyaz	Stomach disorders	Bulbs	Powder	Oral	87	77	88.51	1	89
						Fever								
5	*Allium sativum* L.* (EV-4)*	Amaryllidaceae	H	Garlic	Lasan	Mastitis	Bulbs	Powder	Oral	90	80	88.89	1	89
						Helminthiasis								
6	*Aloe vera* (L.) Burmf*. (EV-17)*	Asphodelaceae	H	Aloe	Kwargandal	Oral infection	Leaves	Powder	Topical	23	13	56.52	1	57
						Wounds								
7	*Amaranthus viridis* L. *(EV-3)*	Amaranthaceae	H	Slender amaranth	Ganhar	Diuretic	Leaves, seeds	Decoction	Oral	20	10	50	1	50
						Tonic								
						Weakness								
8	*Amomum subulatum* Roxb*. (EV-112)*	Zingiberaceae	H	Black cardamom	Kali Elaichi	Indigestion	Fruits	Raw	Oral	80	70	87.5	1	88
						Diarrhea								
9	*Anethum graveolens* L.* (EV-6)*	Apiaceae	H	Dill	Soy	Diarrhea	Seeds	Powder	Oral	81	71	87.65	1	88
						Indigestion								
						Abdominal pain								
10	*Artemisia scoparia* Waldst* &* Kit*. (EV-18)*	Asteraceae	H	Wormwood	Jhahoo	Skin irritation	Leaves	Paste	Topical	22	12	54.55	1	55
						Wounds								
						Cuts								
11	*Arundo donax* L.* (EV-81)*	Poaceae	G	Giant reed	Nerra	Helminthiasis	Leaves	Powder	Oral	26	26	100	1	100
						Diuretic								
12	*Avena sativa* L.* (EV-82)*	Poaceae	G	Oat	Jao	Pneumonia	Seeds	Infusion	Oral	78	76	97.44	1	97
						Weakness								
13	*Azadirachta indica* A. Juss.* (EV-58)*	Meliaceae	T	Neem	Neem	Scabies	Leaves	Infusion	Topical	27	7	26.32	0.63	16
						Skin abscesses								
14	*Baccharoides anthelmintica* (L.) Moench *(EV-19)*	Asteraceae	H	Purple fleabane	kali ziri	Mastitis	Seeds	Raw	Oral	25	17	66.67	1	67
						Allergy								
15	*Bambusa bambos* L.* (EV-83)*	Poaceae	G	Indian thorny bamboo	bans	Helminthes	Leaves	Decoction	Oral	40	38	95	1	95
						Constipation								
16	*Boerhavia procumbens* Banks ex Roxb. *(EV-75)*	Nyctaginaceae	H	Horse-purslane	Itsit	Cough	Roots	Powder	Oral	23	13	56.52	1	57
						Respiratory diseases								
17	*Bombax ceiba* L.* (EV-54)*	Malvaceae	T	Cotton tree	Simbal	Sciatica	Flowers	Powder	Oral	18	5	27.03	0.64	17
						Laxative								
18	*Brassica rapa* L.* (EV-24)*	Brassicaceae	H	Turnip	Gongloo	Eye diseases	Seeds	Oil	Topical	74	64	86.49	1	86
						Skin infection								
						Allergy								
19	*Calotropis procera* (Aiton) W.TAiton* (EV-16)*	Asclepiadaceae	S	Milk weed	Akh	Worm infestation	Leaves, flowers	powder	Oral	72	62	86.11	1	86
						Indigestion								
						Joint pain								
20	*Cannabis sativa* L. *(EV-27)*	Cannabaceae	S	Indian hemp	Bhang	Genitals prolapse	Leaves	Powder	Oral	19	9	47.37	1	47
						Delayed puberty								
						Mastitis								
21	*Capsicum annuum* L.* (EV-102)*	Solanaceae	H	Bell pepper	Hari mirch	Helminthiasis	Fruits	Powder	Topical	70	60	85.71	1	86
						Mastitis								
22	*Cassia fistula* L.* (EV-62)*	Mimosaceae	T	Golden shower	Amaltas	Diarrhea	Fruits	Decoction	Oral	28	8	27.78	0.66	18
						Fever								
23	*Catharanthus roseus* L.* (EV-11)*	Apocynaceae	H	Periwinkle	Sada Bahar	Cuts and wounds	Whole plant	Powder	Topical	11	5	45.45	1	45
24	*Chenopodium album* L.* (EV-28)*	Chenopodiaceae	H	Lamb’s quarter	Bathu	Measles	Whole plant	Powder	Topical	23	13	56.52	1	57
						Wounds								
25	*Cicer arietinum* L.* (EV-41)*	Fabaceae	H	Chickpea	Kala Chana	Piles	Seeds	Powder	Oral	29	19	65.52	1	66
						Weakness								
26	*Citrus limon* (L.) Burmf*. (EV-99)*	Rutaceae	T	Lemon	Nimboo	Mastitis	Fruits	Juice	Oral	22	6	25	0.6	15
						diarrhea								
27	*Cleome viscosa* L.* (EV-25)*	Brassicaceae	H	Tickweed	Jangli hubul	Nasal myiasis	Leaves	Powder	Topical	26	16	61.54	1	62
						Wounds								
28	*Cocos nucifera* L.* (EV-14)*	Arecaceae	T	Coconut	Giri	Infertility	Seeds	Oil	Oral	18	5	28.57	0.68	19
						helminthiasis								
29	*Convolvulus arvensis* L.* (EV-29)*	Convolvulaceae	H	Deer’s foot	Vahri	Galactagogue	Whole plant	Powder	Oral	19	9	47.37	1	47
						helminthiasis								
30	*Cucumis melo var. agrestis* Naudin* (EV-31)*	Cucurbitaceae	H	Wild watermelon	Chibbar	Indigestion	Leaves, Fruits	Raw	Oral	18	8	44.44	1	44
						helminthiasis								
31	*Cuminum cyminum* L.* (EV-7)*	Apiaceae	H	Cumin	Safaid zeera	Gastric ailments	Seeds	Powder	Oral	71	61	85.92	1	86
						Jaundice								
32	*Cuscuta reflexa* Roxb.* (EV-30)*	Convolvulaceae	C	Dodder	Akas bail	Galactagogue	Whole plant	Powder	Oral	20	13	65	1	65
						Astringent								
						Diaphoretic								
33	*Cynodon dactylon* (L.) Pers.* (EV-84)*	Poaceae	G	Bermuda grass	Khabal, Bahm Grass	Inflammation	Whole plant	Paste	Topical	35	33	94.29	1	94
						Wounds								
34	*Cyperus rotundus* L.* (EV-34)*	Cyperaceae	H	Nut grass	Daila	Diuretic	Rhizomes	Powder	Oral	22	12	54.55	1	55
						Helminthiasis								
35	*Dalbergia sissoo* Roxb.ex DC.* (EV-63)*	Mimosaceae	T	Indian rose wood	Tali, Rosewood	Diarrhea	Bark	Decoction	Oral	18	5	29.41	0.7	21
						Bilious disorders								
36	*Datura inoxia* Mill* (EV-103)*	Solanaceae	S	Thorn apple	Datura, Thorn Apple	Delayed puberty	Leaves	Extract	Oral	20	10	50	1	50
						Lice infestation								
37	*Daucus carota subsp. sativus* (Hoffm.) Arcang.* (EV-8)*	Apiaceae	H	Carrot	Gajjar	Placental expulsion	Leaves	Raw	Oral	30	20	66.67	1	67
						Weakness								
38	*Desmostachya bipinnata* (L.) Stapf.* (EV-85)*	Poaceae	G	Tail grass	Dab	Digestive disorders	Leaves	Raw	Oral	29	29	100	1	100
						Fever								
39	*Dodonaea viscosa* Jacq.* (EV-100)*	Sapindaceae	H	Broad-leaf hopbush	Sanatha	Wound	Leaves	Powder	Topical	26	16	61.54	1	62
						Burn								
						Swelling								
40	*Eclipta alba* (L.) Hassk*. (EV-20)*	Asteraceae	H	False daisy	Sofed Banghra	Pneumonia	Leaves	Decoction	Oral	24	14	58.33	1	58
						Weakness								
41	*Eleusine Indica* L.* (EV-92)*	Poaceae	G	Goose grass	Madani	Digestive disorders	Whole plant	Powder	Oral	23	23	100	1	100
						Cough								
42	*Eruca vesicaria* (L.) Cav.* (EV-26)*	Brassicaceae	H	Arugula	Tara Mera	Tick infestation	Seeds	Oil	Topical	67	57	85.07	1	85
						Ectoparasites								
43	*Eucalyptus camaldulensis* Dehnh*. (EV-69)*	Myrtaceae	T	River red-gum	Sofeda	Common cold	Leaves	Decoction	Oral	25	8	30.3	0.72	22
						Gastric ailments								
44	*Eucalyptus globules* Labill* (EV-70)*	Myrtaceae	T	blue gum	Sufaida	Cough	Leaves	Decoction	Oral	25	8	31.25	0.74	23
						Fever								
45	*Eugenia caryophyllata* Thunb* (EV-71)*	Myrtaceae	T	Clove	Loung	Lameness	Fruit	Raw	Oral	57	18	32.26	0.77	25
						Anorexia								
						Pain								
46	*Euphorbia caducifolia* Haines* (EV-35)*	Euphorbiaceae	S	Leafless euphorbia	Danda thor	Snake bite	Leaves	Juice	Topical	19	9	47.37	1	47
						Scorpion bite								
47	*Euphorbia helioscopia* L.* (EV-36)*	Euphorbiaceae	H	Sun euphorbia	Chattri doddak	Febrifuge	Whole plant	Decoction	Oral	20	10	50	1	50
						Galactagogue								
48	*Euphorbia hirta* L.* (EV-37)*	Euphorbiaceae	H	Asthma weed	Doddhi	Fever	Leaves and inflorescence	Powder	Oral	21	11	52.38	1	52
						Common cold								
49	*Euphorbia prostrata* L.* (EV-38)*	Euphorbiaceae	H	Creeping spurge	Doddhi buti	Rashes	Whole plant	Paste	Topical	28	18	64.29	1	64
						Wounds								
50	*Ficus benghalensis* L.* (EV-64)*	Moraceae	T	Banyan fig	Boher, Banyan	Genitals prolapse	Roots, Leaves	Decoction	Oral	25	8	33.33	0.79	26
						Galactagogue								
51	*Ficus carica* L.* (EV-67)*	Moraceae	T	Common fig	Anjeer	Retention of placenta	Leaves, Fruits	Raw	Oral	23	8	33.33	0.79	26
						Digestive disorders								
52	*Ficus religiosa* L.* (EV-65)*	Moraceae	T	Sacred fig	Peepal	Hemorrhagic	Leaves	Decoction	Oral	81	28	34.48	0.82	28
						Septicemia								
						Anestrus								
53	*Foeniculum vulgare* mill *(EV-9)*	Apiaceae	H	Fennel	Soonf	Diarrhea	Seeds	Decoction	Oral	24	14	58.33	1	58
						Indigestion								
						Abdominal pain								
54	*Geranium wallichianum* D. Don Ex Sweet* (EV-47)*	Geraniaceae	H	Cranesbill	Ratan Joot	Abscess	Rhizomes	Powder	Oral	25	15	60	1	60
						Inflammation								
						Galactagogue								
55	*Glycyrrhiza glabra* L.* (EV-46)*	Fabaceae	H	Liquorice	Malati	Cough	Roots	Powder	Oral	19	9	47.37	1	47
						Fever								
						Galactagogue								
56	*Grewia asiatica* L.* (EV-109)*	Tiliaceae	S	Falsa fruit	Falsa	Tonic	Whole plant	Powder	Oral	42	32	76.19	1	76
						Wounds								
						Galactagogue								
57	*Hibiscus rosa sinensis* L.* (EV-55)*	Malvaceae	S	Rose mallow	Gurhal	Galactagogue	Leaves	Raw	Oral	28	18	64.29	1	64
						Fever								
58	*Holarrhena pubescens* Wall. ExG. Don. (*EV-12)*	Apocynaceae	S	Bitter oleander	Kuroo	Heat stress	Bark	Powder	Oral	29	19	65.52	1	66
						Fever								
59	*Hordeum vulgare* L.* (EV-86)*	Poaceae	G	Barley	Joo	Diarrhea	Seeds	Powder	Oral	27	27	100	1	100
						Weakness								
						Cough								
60	*Lagenaria siceraria Molina* Stand L.* (EV-32)*	Cucurbitaceae	C	Bottle Gourd	Kadoo	Worm infestation	Fruits	Raw	Oral	28	21	75	1	75
						Indigestion								
61	*Launaea procumbens* Roxb*. (EV-21)*	Asteraceae	H	Creeping launaea	Pili dodhak	Galactagogue	Leaves	Powder	Oral	22	12	54.55	1	55
						Tonic								
						Lice infestation								
62	*Lawsonia inermis* L.* (EV-52)*	Lythraceae	S	Hina	Mahndi	Genitals prolapse	Leaves	Powder	Oral	19	9	47.37	1	47
						Mastitis								
63	*Lens culinaris* Medik*. (EV-42)*	Fabaceae	H	Lentil	Masoor	Delayed puberty	Seeds	Raw	Oral	22	12	54.55	1	55
						Placenta expulsion								
						Silent estrous								
64	*Linum usitatissimum* L.* (EV-51)*	Linaceae	H	Flaxseed	Alsi	Galactagogue	Seeds	Raw	Oral	82	72	87.8	1	88
						Placenta expulsion								
						Silent estrous								
65	*Mallotus pallidus (*Airy Shaw) Airy Shaw* (EV-39)*	Euphorbiaceae	T	Balik Angin	Kameela	Helminthiasis	Leaves	Powder	Oral	29	10	34.48	1	34
						Control body temperature								
66	*Malva parviflora* L.* (EV-56)*	Malvaceae	H	Cheese-weed	Sonchal	Flatulence	Leaves	Decoction	Oral	23	13	56.52	1	57
						Digestive disorder								
67	*Mangifera indica* L.* (EV-5)*	Anacardiaceae	T	Mango	Aamb	Helminthes	Leaves	Powder	Oral	27	10	35.71	1	36
						Diarrhea								
68	*Medicago sativa* L.* (EV-43)*	Fabaceae	H	Alfalfa	Lusan	Helminthes	Leaves	Powder	Oral	34	24	70.59	1	71
						Diarrhea								
69	*Melia azedarach* L.* (EV-57)*	Malvaceae	T	Chinaberry	Dherak	Anthelmintic	Leaf and fruit	Powder	Oral	27	10	37.04	0.88	33
						Flatulence								
						Fever								
70	*Mentha longifolia* L.* (EV-48)*	Lamiaceae	H	Wild mint	Podina	Diarrhea	Whole plant	Powder	Oral	31	21	67.74	1	68
						Febrifuge								
						Tonic								
						Common cold								
71	*Momordica charantia* L.* (EV-33)*	Cucurbitaceae	H	Bitter gourd	karela	Fever	Whole plant	Infusion	Oral	23	13	56.52	1	57
						Common cold								
72	*Morus nigra* L.* (EV-66)*	Moraceae	T	Mulberry	Kala Toot	Laxative	Leaves, Fruits	Powder	Oral	29	11	37.59	0.9	34
						Tonic								
						Diarrhea								
73	*Myristica fragrans* HOUTT* (EV-68)*	Myristicaceae	T	Fragrant nutmeg	Jaaful	Colic	Fruits, Leaves	Decoction	Oral	26	10	40	0.95	38
						Fever								
74	*Nerium indicum* Mill* (EV-13)*	Apocynaceae	S	Oleander	Kanhera	Helminthiasis	Whole plant	Decoction	Oral	33	16	47.62	1	48
						Gastric ailments								
75	*Nicotiana tabacum* L. *(EV-104)*	Solanaceae	H	Tobacco	Tambaku	Colic	Leaves	Decoction	Oral	71	61	85.92	1	86
						Fever								
76	*Nigella sativa* L.* (EV-94)*	Ranunculaceae	H	Black cumin	Kalonji	Mastitis	Seeds	Decoction	Oral	31	21	67.74	1	68
						Delayed puberty								
						Silent estrous								
77	*Ocimum basilicum* L. *(EV-49)*	Lamiaceae	H	Sweet basil	Niazbo	Diarrhea	Leaves	Decoction	Oral	76	66	86.84	1	87
						Dysentery								
						Helminthiasis								
78	*Olea europaea* L.* (EV-76)*	Oleaceae	T	Olive	Zaitoon	Bone fracture	Fruits	Oil	Oral	39	16	40	0.95	38
						Inflammation								
						Colic								
79	*Oryza sativa* L.* (EV-87)*	Poaceae	G	Rice	Chawal	Diarrhea	Seeds	Powder	Oral	34	31	90.91	1	91
						Dysentery								
						Easy delivery								
80	*Peganum harmala* L.* (EV-74)*	Nitrariaceae	H	Harmal	Hurmel	Gastric ailments	Leaves	Infusion, Decoction	Oral	39	29	74.36	1	74
						Fever								
81	*Pennisetum glaucum* L.* (EV-88)*	Poaceae	G	Pearl millet	Bajra	Galactagogue	Seeds	Powder	Oral	37	37	100	1	100
						Mouth diseases								
						Genitals prolapse								
82	*Phoenix dactylifera* L.* (EV-15)*	Arecaceae	T	date palm	khajoor	Delayed puberty	Fruits	Raw	Oral	60	23	38.46	0.92	35
						Infertility								
83	*Picrorhiza kurroa* Royle ex. Benth.* (EV-101)*	Scrophulariaceae	H	Bitter-root	Kordh	Halitosis	Fruits	Raw	Oral	63	53	84.13	1	84
						Indigestion								
84	*Piper betle* L.* (EV-78)*	Piperaceae	C	Betel vine	Paan	Cough	Leaves	Decoction	Oral	27	20	74.07	1	74
						Fever								
85	*Piper nigrum* L.* (EV-79)*	Piperaceae	C	Black pepper	kali mirch	Cough	Fruits	Powder	Oral	42	35	83.33	1	83
						Fever								
						Mange								
						Mastitis								
86	*Plantago psyllium* L. *(EV-80)*	Plantaginaceae	H	Ispaghula	Isbagol	Digestive disorders	Barks	Powder	Oral	25	15	60	1	60
						Cough								
87	*Portulaca oleracea* L.* (EV-93)*	Portulacaceae	H	Duckweed	Kulfa	Mastitis	Whole plant	Raw	Oral	27	17	62.96	1	63
						Fever								
88	*Prunus dulcis* MilL. D.A.Webb *(EV-97)*	Rosaceae	T	Almond	Badam	Diarrhea	Seeds	Oil	Oral	35	13	37.04	0.88	33
						Abdominal pain								
						Appetizer								
89	*Psidium guajava* L.* (EV-72)*	Myrtaceae	S	Guava	Amrood	Worm infestation	Fruits	Powder	Oral	29	13	45.45	1	45
						Indigestion								
90	*Punica granatum* L.* (EV-53)*	Lythraceae	S	Pomegranate	Anar	Gastric ailments	Fruits	Decoction	Oral	35	15	41.67	0.99	41
						Vermicides								
91	*Ricinus communis* L.* (EV-40)*	Euphorbiaceae	S	Castor oil	Hernoli	Laxative	Seed	Oil	Oral	39	17	43.48	1	43
						Gastric ailments								
						Appetizer								
92	*Rosa indica* L.* (EV-98)*	Rosaceae	S	Rose	Gulab	Abdominal pain	Flower and seed	Decoction	Oral	21	7	33.33	0.79	26
						Mastitis								
						Constipation								
93	*Saccharum spontaneum* L.* (EV-89)*	Poaceae	G	Wild sugarcane	Sarrout	Urinary problems	Leaves	Extract	Oral	37	35	94.59	1	95
						Inflammation								
94	*Sesamum indicum* L.* (EV-77)*	Pedaliaceae	H	Sesame	Til	Delayed puberty	Seeds	Decoction	Oral	53	43	81.13	1	81
						Mastitis								
						Dystocia								
95	*Solanum nigrum* L.* (EV-107)*	Solanaceae	H	Night shade	Makoh	Cough	Whole plant	Powder	Oral	24	14	58.33	1	58
						Fever								
						Worm infestation								
96	*Sonchus asper* (L.) hill* (EV-22)*	Asteraceae	H	Spiny leaved sow thistle	Asgandh, dodak	Galactagogue	Whole plant	Decoction	Oral	31	21	67.74	1	68
						Mastitis								
97	*Syzygium cumini* (L.) Skeels* (EV-73)*	Myrtaceae	T	Jambolan	Jaman	Helminthes	Leaves	Powder	Oral	40	13	31.25	0.74	23
						Diarrhea								
98	*Tamarix aphylla* (L.) HKarst.* (EV-108)*	Tamaricaceae	T	Athel tamarisk	Rukh	Foot and mouth diseases	Leaves	Poultice	Topical	25	8	33.33	0.79	26
						Lice infestation								
99	*Tinosporia cordifolia* (Willd.) Miers* (EV-59)*	Menispermaceae	S	Heart-leaved moon seed	Glow	Foot and mouth diseases	Whole plant	Poultice	Topical	32	13	41.67	0.99	41
						Lice infestation								
100	*Trachyspermum ammi* L.* (EV-10)*	Apiaceae	H	Carom	Ajwain	Galactagogue	Seeds	Powder	Oral	94	84	89.36	1	89
						Appetizer								
						Fever								
101	*Trianthema portulacastrum* L.* (EV-1)*	Aizoaceae	H	Pig weed	Itsit	Helminthes	Whole plant	Powder	Oral	38	28	73.68	1	74
102	*Tribulus terrestris* L.* (EV-114)*	Zygophyllaceae	H	Puncture vine	Gukhroo	Diarrhea	Whole plant	Decoction	Oral	36	24	66.67	1	67
						Urinary problems								
						Colic								
103	*Trifolium alexandrinum* L.* (EV-44)*	Fabaceae	H	Egyptian clover	Barsem	Laxative	Roots	Powder	Oral	30	20	66.67	1	67
						Tonic								
104	*Trigonella foenum-graecum* L.* (EV-45)*	Fabaceae	H	Eugreok	Maithi	Gastric ailments	Seeds	Powder	Oral	25	15	60	1	60
						Diarrhea								
						Delayed puberty								
105	*Triticum aestivum* L. *(EV-90)*	Poaceae	G	Wheat	Gandam	Foot and mouth diseases	Seeds	Powder	Topical	59	57	96.61	1	97
						Weakness								
						Delayed puberty								
						Genital prolapse								
106	*Viola odorata* L.* (EV-110)*	Violaceae	H	Wood violet	Banafsha	Foot and mouth diseases	Whole Plant	Paste	Topical	67	57	85.07	1	85
						Sores								
						Swollen								
						Wounds								
107	*Vitis Vinifera* L.* (EV-111)*	Vitaceae	S	Grape vine	Angoor	Helminthiasis	Seeds	Powder	Oral	28	11	40	0.95	38
						Wounds								
108	*Withania coagulans* Dunal* (EV-105)*	Solanaceae	H	Indian rennet	Paneer dodi	Gastric ailments	Fruits, Leaves	Decoction	Oral	66	56	84.85	1	85
						Worm infestation								
						Mange								
109	*Withania somnifera* (L.) Dunal* (EV-106)*	Solanaceae	H	Winter cherry	Asgandh	Galactagogue	Leaves	Raw	Oral	31	21	67.74	1	67
						Off feeding								
110	*Xanthium strumarium* L*. (EV-23)*	Asteraceae	H	Cocklebur	Chhota Dhatura	Wounds	Leaves	Juice	Topical	39	29	74.36	1	74
						Analgesic								
111	*Zea mays* L.* (EV-91)*	Poaceae	G	Corn	Makai	Weakness	Seeds	Powder	Oral	64	62	96.88	1	97
						Genital prolapse								
						Urinary problems								
112	*Zingiber officinale* Roscoe*. (EV-113)*	Zingiberaceae	H	Ginger	Adrak	Colic	Rhizomes	Powder	Oral	37	27	72.97	1	73
						Fever								
						Indigestion								
113	*Ziziphus jujuba* mill* (EV-95)*	Rhamnaceae	T	Jujube	Bair	Gastric ailments	Fruits, Leaves	Decoction	Oral	26	3	10.61	0.25	3
						Diuretic								
						Helminthes								
114	*Ziziphus nummularia* (Burm.f.) Wight & Arn.* (EV-96)*	Rhamnaceae	T	Wild jujube	Bairi	Wound	Leaves	Paste	Topical	35	4	10.2	0.24	2
						Burn								
						Swelling								

N, Number of informants; Np, Number of informants for specific ailment; FL, Fidelity level; RPL, relative popularity level; and ROP, rank order priority

**Table 2:** Families of medicinal plants used in ethnoveterinary medicine in the riverine areas of Punjab province, Pakistan

Families	Numbers	% contribution
Poaceae	12	10.53
Asteraceae	6	5.26
Euphorbiaceae	6	5.26
Fabaceae	6	5.26
Solanaceae	6	5.26
Apiaceae	5	4.39
Myrtaceae	5	4.39
Malvaceae	4	3.51
Mimosaceae	4	3.51
Moraceae	4	3.51
Apocynaceae	3	2.63
Brassicaceae	3	2.63
Cucurbitaceae	3	2.63
Amaranthaceae	2	1.75
Arecaceae	2	1.75
Asclepiadaceae	2	1.75
Convolvulaceae	2	1.75
Lamiaceae	2	1.75
Liliaceae	2	1.75
Lythraceae	2	1.75
Piperaceae	2	1.75
Rhamnaceae	2	1.75
Rosaceae	2	1.75
Zingiberaceae	2	1.75
Aizoaceae	1	0.88
Amaryllidaceae	1	0.88
Anacardiaceae	1	0.88
Cannabaceae	1	0.88
Chenopodiaceae	1	0.88
Cyperaceae	1	0.88
Geraniaceae	1	0.88
Meliaceae	1	0.88
Menispermaceae	1	0.88
Myristicaceae	1	0.88
Nitrariaceae	1	0.88
Nyctaginaceae	1	0.88
Oleaceae	1	0.88
Pedaliaceae	1	0.88
Plantaginaceae	1	0.88
Portulacaceae	1	0.88
Ranunculaceae	1	0.88
Rutaceae	1	0.88
Sapindaceae	1	0.88
Scrophulariaceae	1	0.88
Tamaricaceae	1	0.88
Tiliaceae	1	0.88
Violaceae	1	0.88
Vitaceae	1	0.88
Zygophyllaceae	1	0.88

The numbers represent the species richness

### Plant parts used and mode of application

**Fig. 3:**
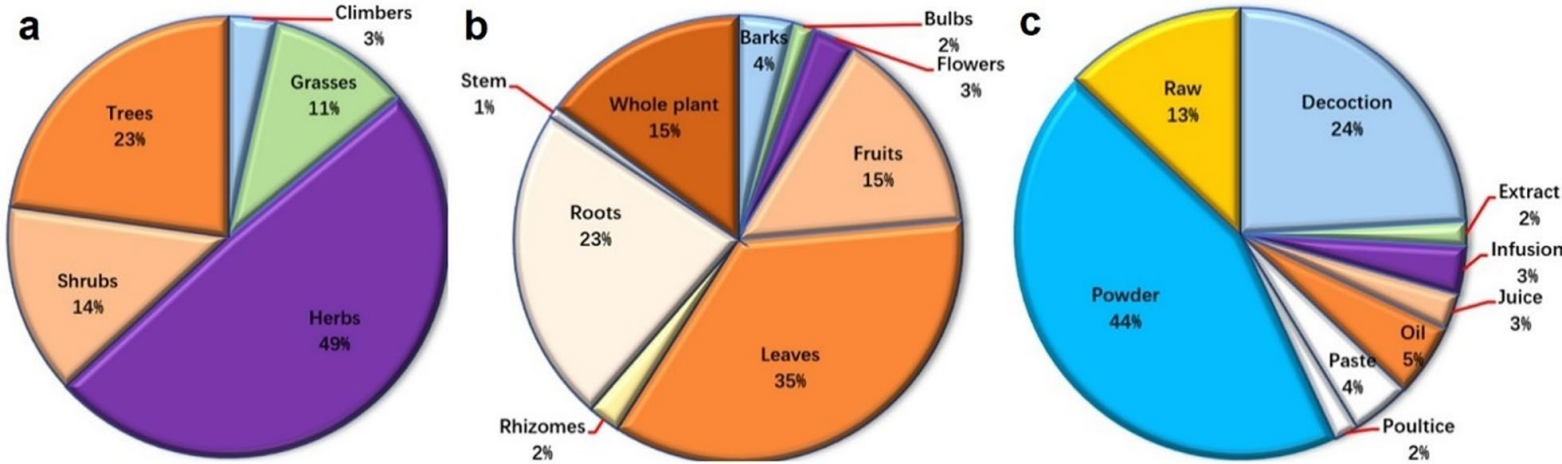
Pie charts shows the percent of **a** growth form distribution, **b** plant parts usage, and **c** method of preparation of medicinal plants in the riverine areas of Punjab province, Pakistan

The leaves of medicinal plant species were the most utilized part in ethnoveterinary treatment, accounting for 35% of all uses, followed by roots (22%), fruits and whole plant (15% each), bark (4%), flower (3%), rhizome, bulb (2% each), whereas other part contributed with only 1% in traditional medicine (Fig. 3). Table 1 lists the growth forms of the reported species. The herbaceous life form contributes the most (49%) of the documented plant species, followed by trees (23%), shrubs (14%), grasses (11%), and climbers (4%) (Fig. 3).

Local residents in the research region prepare recipes to treat many ailments in a variety of forms, including decoction, extract, juice, powder, paste, infusion, poultice, oil, and raw, among others (Fig. 3). Powder (51 applications) was the most often used method of medication preparation, followed by decoction, raw, oil, paste, infusion, extract, and juice (28, 15, 6, 5, 4, and 3, respectively). Extract and poultice, on the other hand, were used in two separate applications.

### Principal component analysis (PCA)

**Fig. 4:**
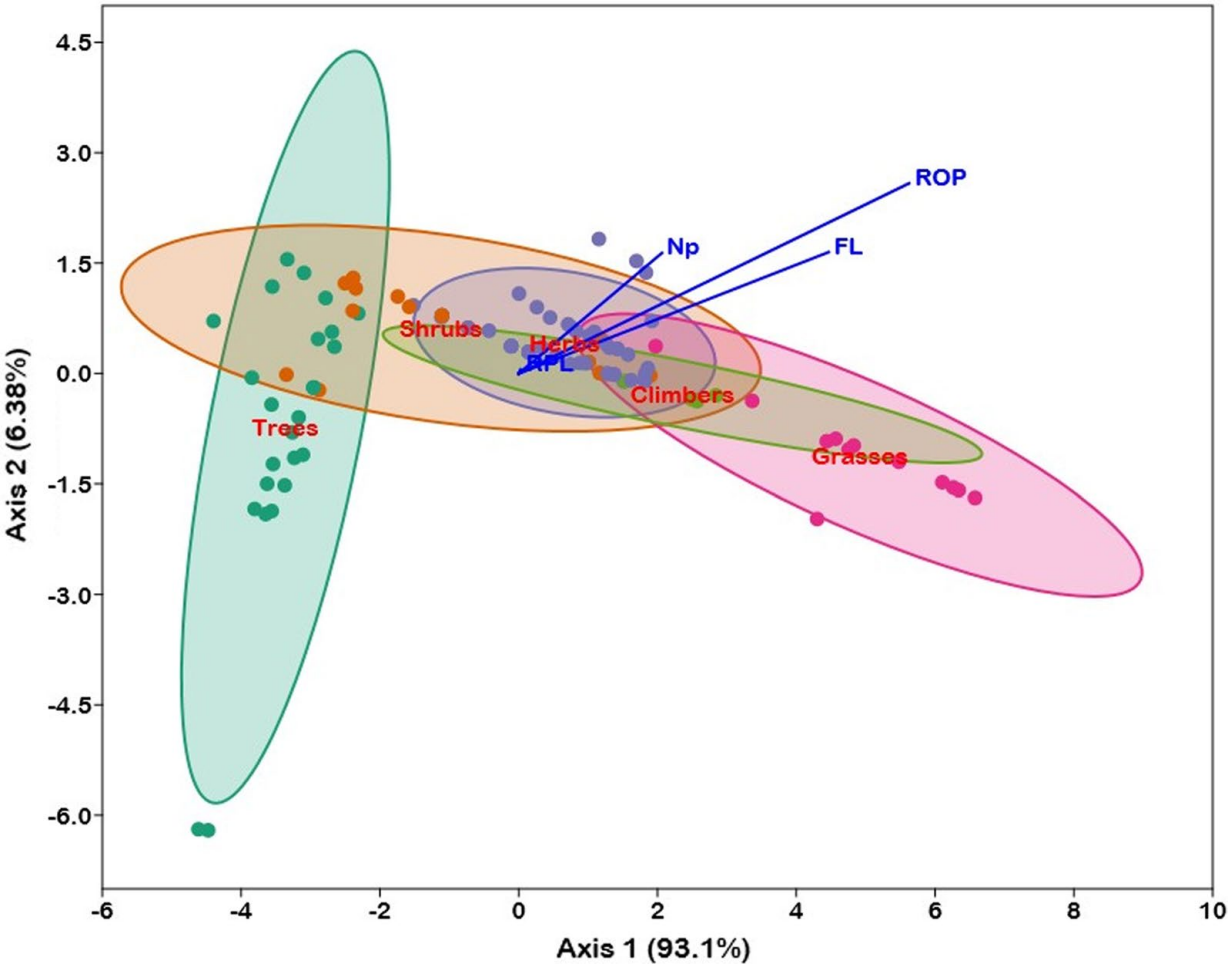
Plot of variables in the PCA conducted with Np (number of informants used particular medicinal plants for specific disease), FL (fidelity level), and ROP (rank order priority) showing the significant differences in the use of different growth form of plant species, i.e., herbs (blue dots), shrubs (orange dots), grasses (pink dots), climbers (golden dots), and trees (green dots). Each line of variables in PCA represents a specific correlation with different growth form of plant species. The length of the blue vector lines represents the total contribution of different growth forms to the analysis. The direction of the blue vector line illustrates the association of variables with each axis. (Vector lines parallel to an axis are significantly connected with that axis.) Correlations between factors are shown by the angles between vector lines

Ethnoveterinary data were subjected to principal component analysis (Fig. 4), which revealed a significant difference in the use of different plant growth forms (herbs, shrubs, trees, grasses, and climbers) for medicinal purposes separated along the axis-1 (*p* 0.05). PC1 and PC2 explained 99.9% of the variance in Np, FL, ROP, and RPL values of medicinal plant species. Grass, herbs, shrubs, and climbers were the most commonly utilized species in the research region, according to PC1-axis factors, whereas trees were rarely employed by indigenous peoples (Fig. 4). For example, five grasses (i.e., *Arundo donax, Desmostachya bipinnata, Eleusine Indica, Hordeum vulgare,* and *Pennisetum glaucum*) are extensively used in the preparation of ethnoveterinary remedies in the research region.

### Category of ailments affecting livestock

Local people used 114 medicinal plants to treat a variety of diseases in livestock (Table 1). The reported ailments were classified into 9 major disease categories on the basis of use reports and number of citations (Table 3). Most use records were in the category endo- and ecto-parasitic ailments (1144 use reports; 1269 citation; 33 plant species; ICF = 0.972). The maximum value of disease cured level (DCL%) was recorded 87.6% for endo- and ecto-parasitic, followed by reproductive disorder (74.9%), fever (74.2%), other health problems (73.4%), gastrointestinal diseases (71.1%), respiratory problems (69.7%), dermatological diseases (66.8%), urinary problems (65.8%), and tonic (63.6%) (Table 3).

**Table 3:** Categories of ailments

Disease category	Taxa used	Use reports	Number of citation	ICF	DCP%
Dermatological	37	897	1287	0.960	66.82
Endo- and ecto-parasitic	33	1144	1269	0.972	87.55
Fever	24	678	881	0.966	74.23
Gastrointestinal	46	1293	1753	0.965	71.14
Other health problem	26	784	1033	0.968	73.38
Reproductive	18	544	702	0.969	74.93
Respiratory disorders	12	260	356	0.958	69.66
Tonic	15	424	643	0.967	63.61
Urinary problems	7	159	231	0.962	65.80

Disease cure percentage (DCP%) and informant consensus factor (ICF)

### Relative popularity level (RPL)

**Fig. 5:**
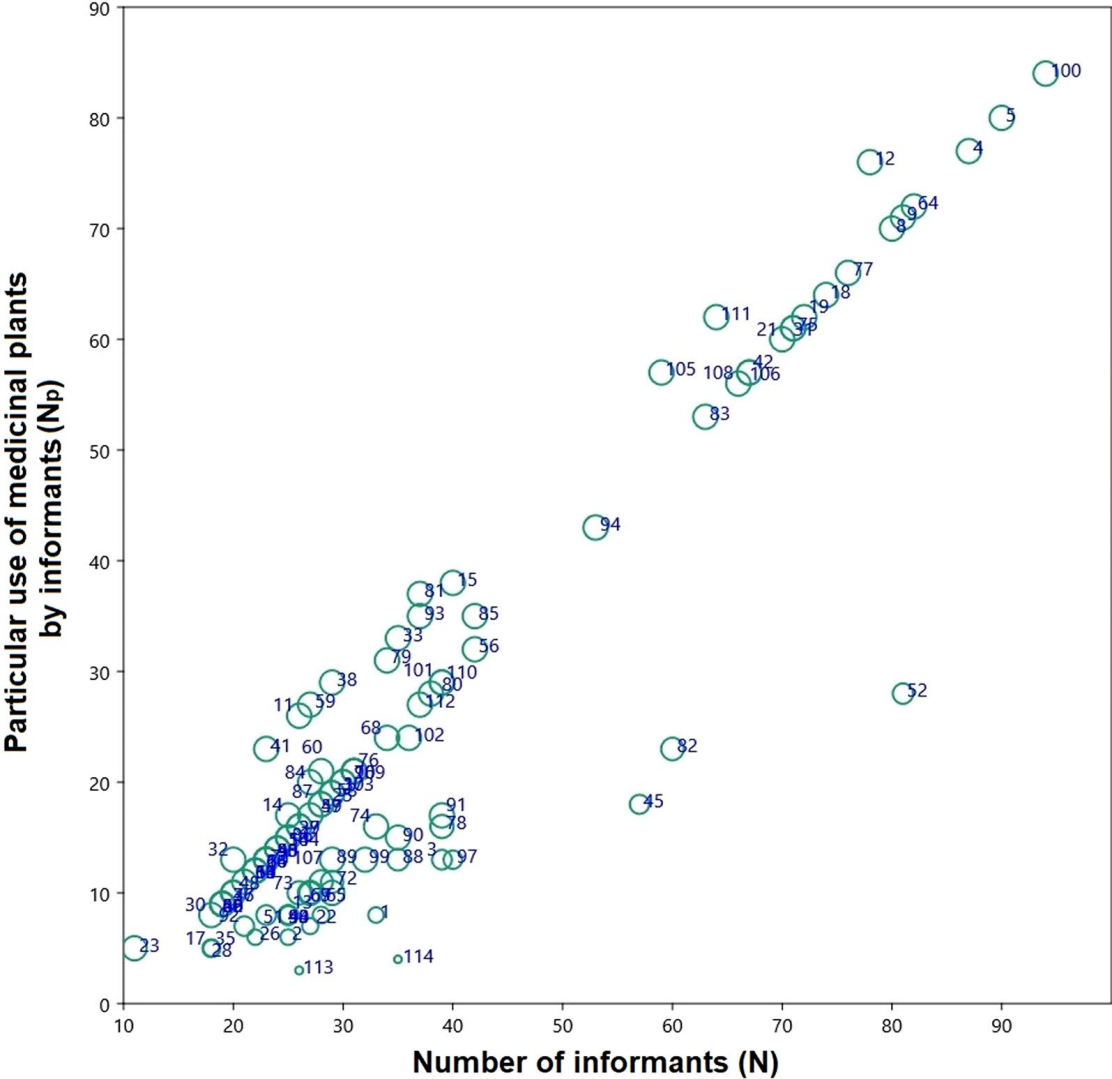
Relation between the number of informants (N) and informants indicated species for certain categories of medicinal use (Np) shows the relative popularity level (RPL) of plant species in the riverine areas of Punjab province, Pakistan**.** Codes are presented in Table 1

The RPL values increase directly with the increase in informants. The RPL value of species varied from 0.22 to 1.00 (Fig. 5). The unpopular species (< 1.00 RPL) in the study area were *Ziziphus jujuba* and *Ziziphus nummularia.*

### Fidelity level and rank order priority (ROP)

**Fig. 6:**
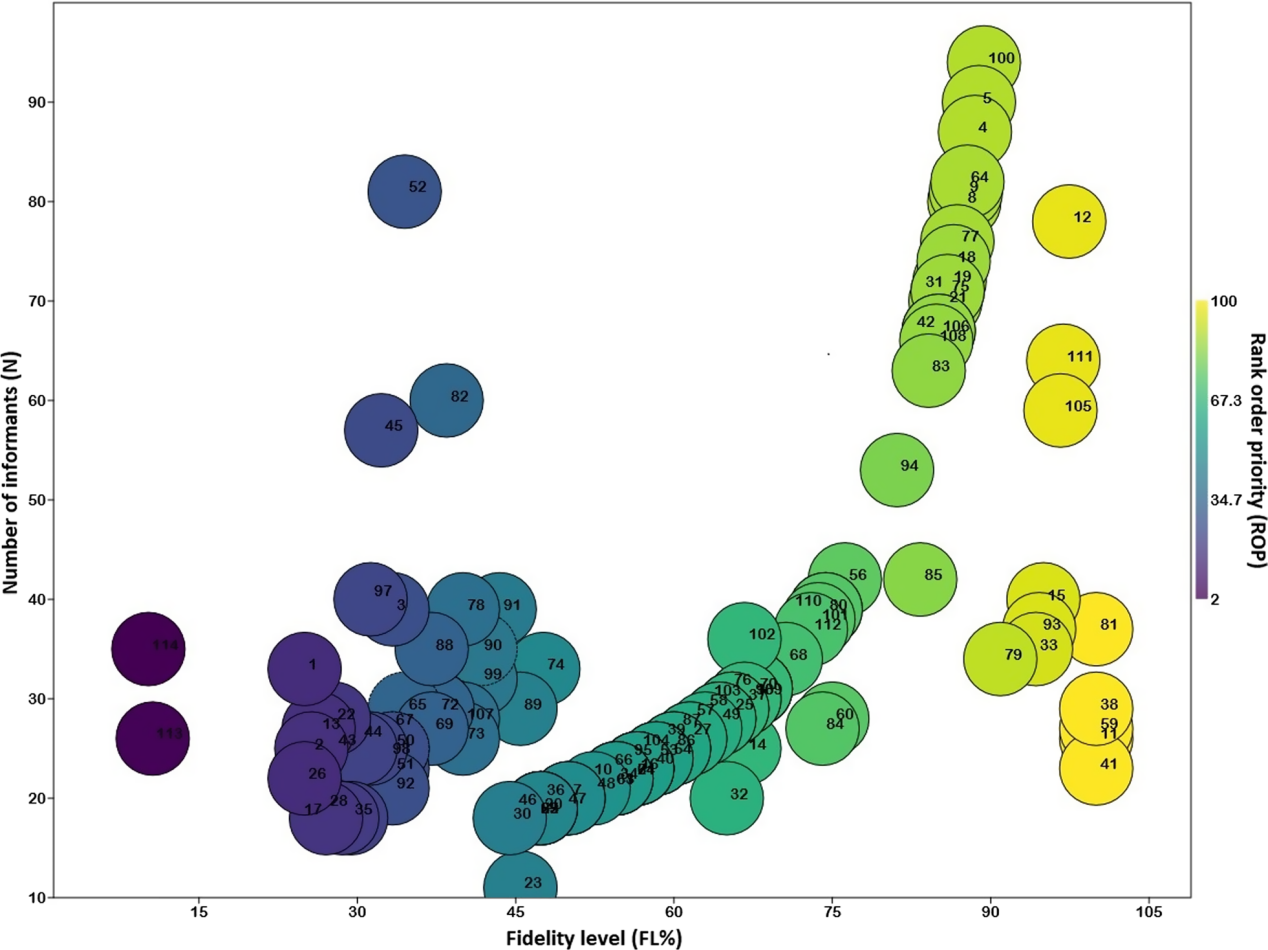
Relation between the number of informants (N) and informants indicated species for certain categories of medicinal use (FL) shows the rank order priority (ROP) of plant species in the riverine areas of Punjab province, Pakistan. Codes are presented in Table 1

The fidelity level is used to discriminate between medicinal plant species that are often utilized by locals to treat various illnesses [51, 52]. The FL of species in this research ranged from 10 to 100% and ROP varied from 2 to 100 (Fig. 6). Five species (*Arundo donax, Desmostachya bipinnata, Eleusine Indica, Hordeum vulgare,* and *Pennisetum glaucum*), which were applied for diuretic, helminthiasis, digestive disorders, fever, cough, worm infestation, indigestion, galactagogue, mouth diseases, and genital prolapsed, had 100% FL and 100 value of ROP (Fig. 6).

## Discussion

### Socio-demographic data

The gathering information on respondents’ sociodemographic characteristics about challenges with livestock nutrition, breeding, and health management, as well as the use of ethnoveterinary approaches, is an important contribution of ethnoveterinary research [53]. Socio-demographic data play a substantial role in interpreting and analyzing the responses received [54].

Traditional healing is often a gendered activity in which both men and women take part [55]. In the study area, female interviewers were uncommon (Fig. 2). This is due to the fact that fewer women work in livestock management and cattle husbandry. Most women were not permitted to debate or share indigenous knowledge with outsider males. Another ethnoveterinary research from Pakistan [7, 56] and overseas revealed a similar pattern [57–59]. Our findings contradict the findings of an ethnoveterinary research conducted in Neelum Valley, Kashmir Himalaya, Pakistan. They found that women are more in number than males and had better knowledge about ethnoveterinary medicine practice [60]. In general, gender disparities in ethnoveterinary medicine knowledge can be attributed to experience and cultural exposure with therapeutic plants. Men are the primary collectors of medicinal plants growing wild, which could explain our findings.

Compared to younger participants, older responders, especially those over 60 years old, are more numerous and have a high level of traditional knowledge (Fig. 2). Similar studies conducted in Pakistan and other countries found that respondents who were older were more numerous and possessed more traditional knowledge than those who were younger [61–64]. Community elders are often the ones who have the most information about therapeutic plants, according to Nolan and Turner [65]. Because community elders are occupied with family responsibilities including financial, health, and training, they no longer pass on their expertise to the following generation. The understanding of the usage of therapeutic herbs is vanishing as a result.

Indigenous knowledge regarding the use of medicinal plants was more common among illiterate people (36.9%), and knowledge was diminishing within education. These studies results are in agreement with other ethnobotanical studies carried out in Pakistan [63] and Morocco [66]. Because of their greater exposure to modernization, educated persons are found to be less knowledgeable about the use of medicinal plant in ethnoveterinary practices than uneducated people in Punjab, Pakistan. Furthermore, because of the low literacy rate in the study region, the residents are significantly reliant on medicinal plants for a few uses to sustain their needs. Education reform is also critically required to preserve folklore and traditional knowledge, as well as prioritized majors such as humanities and social sciences [67].

In our study, traditional medical practitioners (TMPs) with more than 20 years of experience held the most knowledge about ethnoveterinary practices. Similar findings were found in other areas, e.g., in Turkey [68, 69] and Bangladesh [70]. Plant species were harvested by TMPs during appropriate times of seasons. Some of the locals also grew medicinal plants and sold them, typically for modest costs, to herbalists or in nearby marketplaces. Many TMPs recorded their herbal formulations in writing, but they typically did not divulge this information to the public in order to limit the number of healers. They preferred to transfer their knowledge verbally either to their close relatives, i.e., wife, son, daughter, and brother, or to their helpers and students. The usage of medicinal herbs was frequently found to be a daily ritual in many homes, where such knowledge was passed down to the younger generation simply by observing the elders practice. The most frequent transfer of cultural and traditional knowledge, like in other communities across the world, was found to be from parents to children, especially boys [71–76]. It is also critical to protect traditional knowledge and local flora by promoting awareness among local communities about the value of their knowledge and plants [77].

### Pharmacological assessment

Medicinal plants of this area are very famous, and effectiveness of medicinal plants is seen as associated with the climate of the area (arid to semiarid), which might lead to an increase in the concentration of bioactive molecules [78]. We found that local people used more grasses, climbers, and herbs to treat various livestock ailments, while trees are less commonly used in the treatment of various livestock ailments (Fig. 4). According to Harun et al. [79], grasses are chosen over other therapeutic herbs and plants for treating bovine illnesses in agricultural areas such as Punjab because they are abundant, pleasant, and easy to collect. Here, we addressed the pharmacological uses of several species against some specific diseases in the research region to better assess and estimate quantitative data obtained concerning the use of species in ethnoveterinary remedies.

### Medicinal uses of plants in ethnoveterinary medicine (EVM)

**Fig. 7:**
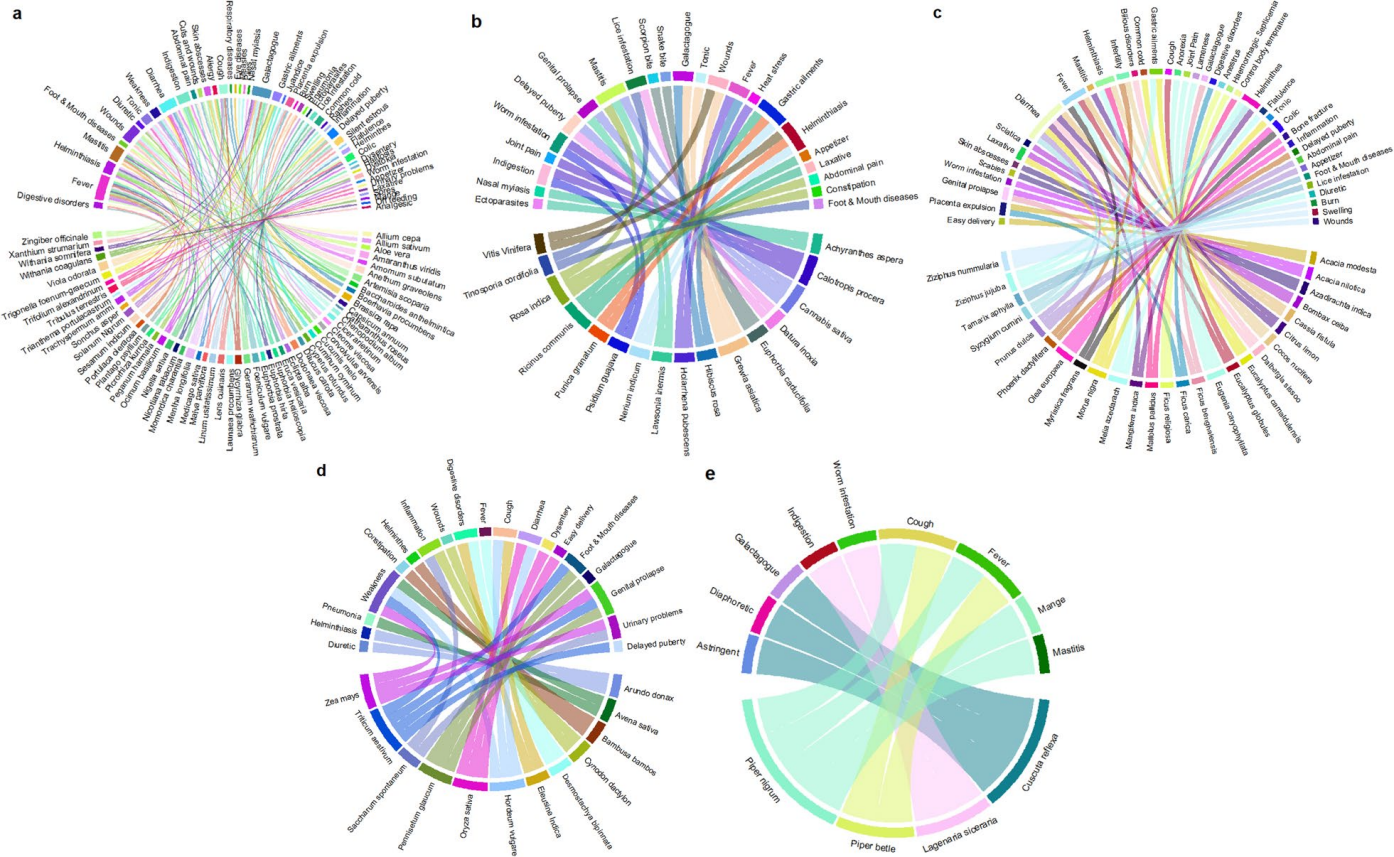
The chord diagram represents the usage of medicinal plants in ethnoveterinary remedies. Various plant growth forms **a** herb, **b** shrubs, **c** trees, **d** grasses, and **e** climbers used for treating specific ailments in the riverine areas of Punjab province, Pakistan

Herbs provided the majority of the components used in remedies (Fig. 7a). People have more opportunities to experiment and learn about therapeutic applications because herbs are frequently the most commonly used species [80]. Herbs have applications in gynecological, surgical, and bovine mastitis interventions as well as a variety of other infections as acaricidal and anthelmintic treatments [81]. According to Silva et al. [82], herbaceous plants often have a greater amount of bioactive chemical compounds than shrub or tree species.

In this study, the FL% of herbs ranged from 89 to 44% (Fig. 6). The most used herbs were *Allium sativum* (mastitis)*, Allium cepa* (stomach disorders), *Trachyspermum ammi* and *Linum usitatissimum* (galactagogue), *Amomum subulatum* (indigestion), *Anethum graveolens* and *Ocimum basilicum* (diarrhea), had FL more than 85% (Table 1 and Fig. 7a). *Allium sativum* extract was found to significantly inhibit the activity against bacterial strains [83]. When compared to other anti-mastitis plants, *A. sativum* had the greatest concentration of antioxidants and was identified as a feasible choice for the development of innovative veterinary medications with minimal cost and less adverse reactions. According to Mehlhorn et al. [84], sheep with gastrointestinal cestodes and nematodes were fed specific extracts of *Allium cepa* (onion) and *Cocos nucifera* (coconut) along with polyethylene glycol propylene-carbonate or milk powder for eight days. When compared to untreated animals, they gained significant weight and greatly diminished the worm infestation. In India, “National Dairy Research Institute” examined the content of estrogen in several plants (including *T. ammi*) that are traditionally used to enhance milk production in cattle and sheep [85]. The phytoestrogens found in *T. ammi and L. usitatissimum* seeds, referred to as galactagogues [86, 87]. According to Kaur [87], the dried *T. ammi* seed exhibited the second-highest total phytoestrogen concentration (473 ppm) among the eight plants studied (total concentrations of estrogen, 131–593 ppm). The fruits of *Amomum subulatum* are used to treat gastrointestinal ailments in the Ayurvedic medicine (i.e., desiccant, stomachic, digestive, resolvent, and carminative) [88]. The ability of petroleum ether soluble fractions and essential oils of *A. subulatum* to prevent aspirin and ethanol-induced gastrointestinal lesions was investigated in rats. Both pharmaceutical components effectively reduced gastrointestinal lesions. [89]. *Anethum graveolens* powder was used to treat irritable bowel syndrome, and after two weeks, all IBS symptoms improved without any side effects [90]. Sweet basil (*Ocimum basilicum*) is a popular and widely used spice that has been found to have antioxidant, antibacterial, and anti-diarrheal properties [91].

Shrubs are used to prepare majority of recipes (Fig. 7b) as they are accessible all year [92]. Indigenous knowledge of the medicinal and ethnoveterinary uses of shrubs can benefit livestock farmers economically [93]. Although the current scientific world is unfamiliar with indigenous knowledge and traditional uses of shrubs in ethnoveterinary practice [94], *Calotropis procera* (Aak in Punjabi) is widely used in traditional medical system of Pakistan [95], as an laxative, anthelmintic, expectorant, anti-inflammatory, and diuretic [96, 97]. The latex-rich blooms of this plant are widely recognized for both their therapeutic and poisonous characteristics, which include their impact on worm infestation. In Pakistan, the most common methods for administering *C. procera* flowers as an anthelmintic to small ruminants are crude powder or decoction combined with honey [98]. Chemical components can fluctuate significantly across plants because of environmental or genetic variations, plant growth phases at harvest, and storage methods [99]. According to in vitro investigations, “crude methanolic” and “crude aqueous” extracts of *C. procera* blooms were shown to exhibit antihelmintic effects on live *Haemonchus contortus,* as proven by death or transitory paralysis. According to in vivo investigations, *C. procera* flower extracts (“crude powder,” “crude aqueous,” and “crude methanolic”) were administered to sheep that were naturally infected with a number of gastrointestinal worms, resulting in the reduction of nematodes egg percent [95].

Ethnoveterinary trees are plants that are used to cure common cattle ailments (Fig. 7c). In most situations, infusions or decoctions of tree barks are drank or used topically to cure common cattle ailments [100]. Leaf extracts of many tree species are used in EVR to treat helminth infections [101]. Ruminant production is hampered by parasitic illnesses on a global scale, which results in significant economic losses from stunted development, weight loss, decreased food consumption, decreased milk output, poor fertility, and, in severe infections, high mortality rates. From the perspective of parasite management and their minimal environmental impact from residues compared to commercial anthelmintics, the use of plant-based veterinary medication with anthelmintic qualities appears to be a successful option. For instance, the helminth infections-treating plants *Melia azedarach, Mangifera indica, Mallotus pallidus,* and *Syzygium cumini* exhibited FL values of 37.0, 35.7, 34.5, and 31.3, respectively. Szewczuk et al. [102] found that extracts obtained from *M. azedarach* drupes were shown to be more efficacious against tapeworms and hookworms which cause gastrointestinal diseases in goats and sheep.

Grasses are used not only for hay and pasture, but they also have an essential role in the treatment of a number of cattle ailments (Fig. 7d). The fidelity level (FL%) is used to identify between medicinal plant species that are often utilized by locals to treat various illnesses. In this study, the faithfulness level (FL%) of grass species ranged from 100 to 90%. Locals in rural Punjab utilize grasses extensively for ethnoveterinary uses [69]. The fidelity level (FL%) is used to identify medicinal plant species that are often utilized by locals to treat various illnesses. FL (%) of grasses in this research ranged from 90 to 100%. Locals in rural Punjab employ grass species extensively for ethnoveterinary purposes. According to Harun et al. [79], certain stomach issues have been reported to be treated by *Bromus japonicus, Cynodon dactylon, Eragrostis minor, Desmostachya bipinnata, Eleusine indica,* and *Phragmites australis*, whereas microbial infections in cattle can be treated by *Arundo donax, Brachiaria ramosa, Chrysopogon zizanioides, Panicum antidotale,* and *Sorghum bicolor* Furthermore, rural communities all over the world use grass species as a key source of domestic animal feed and as a medication to cure a variety of illnesses in both humans and animals.

Grasses are particularly important in health care because they contain physiologically active compounds such as alkaloids, flavonoids, and saponins [103]. In our investigation, 100% fidelity level was observed for five species to treat ailments, i.e., *Arundo donax* for the treatment of helminthiasis, *Desmostachya bipinnata* for ailments of digestive disorders, *Eleusine Indica* for therapy of cough, *Hordeum vulgare* for treatment of diarrhea, and *Pennisetum glaucum* for treatment of galactagogue. According to Sharatkumar et al. [104], *A. donax* crude powder reduced the count of fecal egg by 50.5% in sheep naturally infected with gastrointestinal parasites. Antihelmintic activities of *A. donax* extracts (about 55% effectiveness) against cattle gastrointestinal parasites (*Ascaris, Oesophagostomum, and Paramphistomum*) [105]. The root of *D. bipinnata* has been utilized in Ayurvedic medicine to treat digestive problems. The *D. bipinnata* alcoholic extract substantially decreased the faces weight, and it also delayed the flow of charcoal meal through the digestive tract [106]. *Eleusine indica* aerial parts infusion is used to treat respiratory illnesses such as pneumonia and influenza that induce airway irritation [107]. Pretreatment with crude extract of *E. indica* inhibited pulmonary neutrophil activation by 98% in rats exposed to lipopolysaccharide aerosols from Gram-negative bacteria. These findings may clarify the widespread usage of *E. indica* to treat inflammatory diseases of the airways. Sena et al. [108] reported that rice starch mixed with barley (*Hordeum vulgare*) flour in 3: 1 ratio had an effective supportive therapy in diarrhea in calves. Arogundade et al. [109] reported that *P. glaucum* grains have been used to improve lactation. According to the histopathological analysis performed on rats, *P. glaucum* extract increased milk production and emptying in a similar manner to domperidone. The extract of grains demonstrated potential as a galactagogue that may be used not only in humans but also in the dairy sector to improve milk production.

Climbers have an essential role in human health care as self-medication [110]. Climbers have been employed in a variety of medical procedures for millennia, and traditional medical practitioners (TMP) have reported excellent medical outcomes, as some of the medications have been discovered to cure numerous life-threatening and serious chronic conditions. Several of the climbers identified in the literature of Ayurveda as medicinally useful have been validated by scientists and shown to have fascinating pharmaceutical properties such as antiulcer, anti-diabetic, anti-arthritic, and anti-tumor [111]. Ajaib et al. [112] reported the use of 36 climber plants for food, medicines, and livestock by the native inhabitants of AJK, Pakistan. Another research from India describes the use of 26 climbers as EVR for the treatment of endoparasites in goats [113]. The highest FL of *Piper nigrum*, a climber species used to cure cough (Fig. 7e), was 83% in our research (Table 1). *P. nigrum* fruits are not only a beloved spice, but also a valuable medicinal agent that treats a variety of disorders such as cold, asthma, cough, and respiratory disorders [114]. According to Khawas et al. [114], the extract of *P. nigrum* fruit showed antitussive activity in guinea pigs to various extents that is connected to pectic and piperine polysaccharide with type II arabinogalactan sidechains.

### Major ailments affecting livestock

Endoparasites (*Haemonchus* spp.*, Paramphistomum* spp., *Toxocara* spp., *and Coccidial oocysts)* and ectoparasites (ticks, lice, and mites) are serious illnesses affecting livestock in the research region. Studies carried out in various countries show that the average extensiveness of gastrointestinal nematode infection (endoparasites) ranges from 61.96% in Mexico [115], 56% in Canada [116] to as much as 90% in Belgium and the Netherlands [117]. Wide distribution of ecto- and endoparasites in cattle is a serious problem in the sustainability of a farm, due to the negative impact on animals’ health and productivity [118]. Parasitic invasions are the major cause of production losses in dairy cattle herds including losses in milk production, decreased growth performance, impaired reproduction, and poor welfare [119, 120]. The signs of parasitosis are not specific and often pass without any noticeable signs. The action of parasites leads to chronic disease and economic losses long after the invasion has ceased [121].

The hot, humid weather of Punjab province’s riverine regions fosters the creation and growth of endoparasites (*Haemonchus* spp*.* and *Toxocara* spp*.*) and ectoparasites (ticks, lice, and mites), resulting in aggressive parasitism. This conclusion validates Hassan et al. [122] findings, which found that Bangladesh’s hot, humid environment favors the formation and survival of ectoparasites and endoparasites, as well as parasitic violence. The signs of an ecto-parasitic infection are visible to the naked eye. The larvae or adults of the parasite are visible on the animal’s body. The animals become restless as a result of the extreme itching, and the condition of their hair and skin deteriorates. These animals are less productive because they have less time to relax, ingest, and chew food while fending off insects [123]. Endo-parasitic infections cause general weakness and a decrease or inhibition of weight gain. It is brought on by digestive issues, malabsorption, or diarrhea. Sick animals have pale mucous membranes due to anemia. Reduced milk supply and changes in milk nutritional composition are markers of cow parasite infection [124, 125], as are reproductive difficulties such as embryo death and miscarriage. The severity of symptoms is determined by the parasite type, the level of infection, the food ration, the environment, and the animal’s overall health. In many cases, people are more vulnerable to bacterial and viral diseases [118]. Indigenous peoples of Punjab used different medicinal plants to treat endo- and ectoparasites infection such as, *Achyranthes aspera, Allium sativum, Arundo donax, Bambusa bambos, Calotropis procera, Capsicum annuum, Cleome viscosa, Cocos nucifera, Convolvulus arvensis, Cucumis melo, Cyperus rotundus, Datura inoxia, Eruca vesicaria, Ficus religiosa, Lagenaria siceraria, Launaea procumbens, Mallotus pallidus, Mangifera indica, Medicago sativa, Melia azedarach, Nerium indicum, Ocimum basilicum, Piper nigrum, Psidium guajava, Punica granatum, Solanum Nigrum, Syzygium cumini, Tamarix aphylla, Tinosporia cordifolia, Trianthema portulacastrum, Vitis Vinifera, Withania coagulans,* and *Ziziphus jujuba* (Table 1).

### Traditional knowledge for animal health management

Traditional knowledge plays a significant role in human–environment interactions in local ecosystems [126]. Indigenous peoples have relied on nature for survival for millennia, resulting in a strong connection with their environment and a sense of their surroundings based on observation and experience [127]. Local communities have developed an awareness of their environment, which has shaped and sustained intergenerational livelihoods such as livestock rearing, crop cultivation, and animal husbandry [126]. We noticed that locals have more knowledge about using grasses to cure various livestock diseases. Majeed et al. [35] found that the inhabitants of Punjab province have a strong connection to their surrounding plant diversity and extensive traditional knowledge about the medicinal uses of grasses to treat a number of animal health issues. Due to a shortage of veterinary clinics and extension services, high drug costs, and the efficacy of ethnoveterinary treatments, many farmers have preferred to employ their traditional knowledge for animal health management. In general, these approaches are low cost, locally available, and sustainable, particularly in times of climate change and fluctuation [128]. Promoting ethnoveterinary heritage in rural development programs in Pakistan can improve animal welfare and food quality [21].

Research and scientific validation of traditional knowledge on EVR are thus critical for increasing their application in animal health management. The knowledge of experienced elderly people and traditional health practitioners should be used to gather information on these methods so that future generations might enjoy the same advantages [53]. For example, the forms in which the medicine must be administered for a certain ailment, and this knowledge must be preserved for the benefit of future generations.

## Conclusion

To the best of our knowledge, traditional medicinal applications of 114 plant species that are used to cure a range of animal illnesses in the research region have been described. *Arundo donax, Desmostachya bipinnata, Eleusine Indica, Hordeum vulgare,* and *Pennisetum glaucum* showed a 100% FL value when used to treat diuretics, helminthiasis, digestive problems, fever, cough, worm infestation, indigestion, galactagogue, oral infections, and genital prolapse. Nonetheless, grasses are particularly important in medicine because they contain physiologically active substances such as flavonoids, alkaloids, and saponins. Endoparasites (*Haemonchus* spp.*, Paramphistomum* spp., *Toxocara* spp., *and Coccidial oocysts)* and ectoparasites (ticks, lice, and mites) are serious illnesses affecting livestock in the research region. The maximum value of disease cured level (DCL%) was recorded 87.6% for endo- and ecto-parasitic ailments in the study area.

The abundance of indigenous knowledge on EVR is a significant validation for expanding the process to collect records from other regions of Pakistan in order to gather important information about current plant-based veterinary practices and incorporate them into the official R&D system of veterinary science. Documenting the traditional knowledge has several uses, including safeguarding it for future generations, making it available to the public, and utilizing it as a starting point for more study and conservation initiatives. This EVR knowledge is useful for contemporary pharmaceutical research since it may open up opportunities for the identification of new compounds with significant therapeutic potential in the future. Important toxicological studies would be necessary to guarantee the continuous and secure use of the presented EVR practices.

## Data Availability

All the data are presented in tables and figures in the article or as a supplementary material, and further inquiries can be directed to the corresponding author.
